# Multiple biomarker responses in caged benthic gastropods *Bellamya aeruginosa* after in situ exposure to Taihu Lake in China

**DOI:** 10.1186/s12302-018-0164-y

**Published:** 2018-09-11

**Authors:** Qian Li, Meng Wang, Lei Duan, Yanling Qiu, Taowu Ma, Ling Chen, Magnus Breitholtz, Åke Bergman, Jianfu Zhao, Markus Hecker, Lingling Wu

**Affiliations:** 1 State Key Laboratory of Pollution Control and Resource Reuse, College of Environmental Science and Engineering, Tongji University, Shanghai, 200092 China; 2 College of Biology and Environmental Sciences, Jishou University, Jishou, 416000 China; 3 Department of Environmental Science and Analytical Chemistry, Stockholm University, Svante Arrhenius väg 8, SE-11418 Stockholm, Sweden; 4 Swedish Toxicology Sciences Research Center (Swetox), Forskargatan 20, 15136 Södertälje, Sweden; 5 School of the Environment & Sustainability and Toxicology Centre, University of Saskatchewan, 44 Campus Drive, Saskatoon, SK S7N 5B3 Canada

**Keywords:** Freshwater sediment, *Bellamya aeruginosa*, Caged organisms, Biomonitoring

## Abstract

**Background:**

Freshwater sediments have been recognized as a long-term sink and potential source for environmental pollutants released into the aquatic ecosystems. In this study, the sediment quality of Taihu Lake, which is susceptible to anthropogenic contamination, was assessed by a combination of chemical analytical and biological end points. Specifically, the snail *Bellamya aeruginosa* was caged in situ at two locations representing different pollution levels for different exposure times (7, 14 and 21 days). At each of these time points, biochemical parameters, i.e., phase I biotransformation enzymes ethoxyresorufin-*O*-deethylase (EROD), the antioxidant enzymes superoxide dismutase and catalase, reactive oxygen species, protein carbonyl content and lipid peroxidation, were evaluated in the hepatopancreas of snails. In addition, surface sediments were collected for analysis of contaminants of concern, including inorganic pollutants, organochlorine pesticides, polychlorinated biphenyls and polybrominated diphenyl ethers.

**Results:**

Chemical analyses revealed that sediments from Taihu Lake were contaminated with trace elements and organic pollutants. Concentrations of trace elements (Cu, Ni and As) and organochlorinated pesticides (4,4′-DDE) exceeded their corresponding threshold effect level according to the sediment quality assessment values for freshwater ecosystems in Canada, indicating that adverse biological effects may occur. All biomarkers, except EROD activity, were induced in snails during all exposure times. The integrated biomarker response index (IBR) indicated that during the initial exposure phase (7 days), *B. aeruginosa* were subjected to significant environmental stress, which diminished during later sampling time points.

**Conclusions:**

Results showed that IBR correlated well with the levels of environmental contaminants, demonstrating the applicability of this biomonitoring approach to complex environmental exposure scenarios.

**Electronic supplementary material:**

The online version of this article (10.1186/s12302-018-0164-y) contains supplementary material, which is available to authorized users.

## Background

In recent years, water quality of many freshwater lakes has been shown to progressively deteriorate with the rapid economic growth in many developing countries. Main contributors to the contamination of lake ecosystems are runoff from agricultural activities and industrial and municipal effluents [1, 2]. Sediments are important components of the aquatic environment and constitute a particular concern from an environmental toxicology perspective, as they can retain persistent and toxic chemicals at levels many times greater than concentrations typically found in the water column [3]. Contaminated sediments may pose direct threats to benthic biota and to organisms that feed on the benthos [4, 5]. Therefore, characterization of sediment quality is of great relevance for the risk assessment of contaminant to aquatic ecosystems and potential human health. There are several tools for the assessment of sediment quality, including routine chemical analyses and sediment toxicity biological assays or tests [6]. Toxicity tests have been proven to be highly useful and relevant as they can often be done more quickly and inexpensively compared to chemical analyses and can provide insights into the effect of sediment-bound chemicals on organisms [7].

Field toxicity tests using caged organisms present numerous advantages over laboratory toxicity testing and indigenous community surveys [8]. In situ caging experiments involving organisms such as fish [9, 10], bivalves [11–13], crustaceans [14, 15], gastropods [16, 17], polychaetes [18] and oligochaetes [19] capture the complex interactions and more realistic exposure conditions compared to laboratory tests [8]. Furthermore, compared with studies of native populations, the use of caged organisms affords numerous advantages such as the ability to select specific species and developmental stages, to control exposure duration and to focus on specific locations as animals are prevented from moving into or out of sites [20]. In addition, the use of caged organisms can minimize the influence of adaptive mechanisms, which may have evolved in resident organisms over time under long-term chronic exposure conditions and would lead to the underestimation of pollution [14].

Benthic gastropods are essential members of aquatic systems and relatively sensitive to contaminants [21]. *Bellamya aeruginosa* (synonym: *Sinotaia aeruginosa*; Gastropoda, Caenogastropoda, Viviparidae) is commonly found in various freshwater habitats of lakes (including Taihu Lake), reservoirs, rivers, streams, ditches and ponds throughout China. This species is a dominant community member of freshwater aquatic systems [22] and it is therefore an ecologically important representative of benthic macroinvertebrates. As a deposit-feeding gastropod, *B. aeruginosa* ingests sediment particles, organic detritus, algae and bacteria on the surface of sediments or other substrates [23] and, therefore, it is highly susceptible to be exposed to sediment-associated contaminants. This species has been found to accumulate contaminants and to be sensitive to sediment-borne pollutants at the biochemical level [22, 24]. *B. aeruginosa* is a primary food item of the black carp (*Mylopharyngodon piceus*), which is also consumed by humans. Therefore, it represents a key organism involved with the transfer of contaminants through the food web in Chinese surface waters and, thus, it can be recommended as a key organism in the assessment of contaminant risks to ecosystems in China.

The impacts of sediment contamination on biota can be determined by the measurement of biomarkers [18]. Biomarkers can provide sensitive and biologically relevant information and can serve as an integrative measure of the effects of mixtures of chemical stressors [25, 26]. Another advantage of using biomarkers lies in their potential to anticipate damage at higher levels of biological organization before ecological disruption occurs [27]. Since there is no single biomarker that can reflect the overall health status of organisms, it has been recommended that a battery of biomarkers should be used to understand the adaptive responses to environmental conditions [27, 28]. Common biomarker end points that are often observed under complex environmental exposure scenarios include impacts on metabolic systems involved in detoxification of xenobiotics as well as antioxidant responses and oxidative stress. The activity of EROD (ethoxyresorufin-*O*-deethylase) represents the activity of the phase I biotransformation enzyme, cytochrome P4501A (CYP1A), which is essential for detoxification and excretion of chemicals. Measurements of ROS (reactive oxygen species) production and antioxidant defense in invertebrates are also applied widely for monitoring of pollution of aquatic environments [29, 30]. Many pollutants exert their effects through redox cycling, resulting in the production of ROS [10]. ROS can be detoxified by the antioxidant defense system including the SOD (superoxide dismutase) and CAT (catalase) enzymes. The imbalance between production of ROS and antioxidant defenses may lead to oxidative stress manifested as oxidative damage of lipids and proteins [31]. To integrate the responses of different biomarkers into a single value or graph, the methodology of the integrated biomarker response (IBR), described by Beliaeff and Burgeot [32], is widely used in field and laboratory studies [33, 34]. The second version for the index (IBRv2) was proposed based on the reference deviation concept to avoid the weakness of the first version by Sanchez et al. [35].

Taihu Lake, located in the delta region of the Yangtze River, is the third largest freshwater lake in China and an important drinking water source for surrounding cities [36, 37]. With rapid economic development and population increase in the Yangtze River Delta, untreated wastewater from plants and mills was directly discharged into rivers during the past decades and ultimately entered the lake, resulting in serious pollution of Taihu Lake [38, 39]. Contaminants commonly found in Taihu Lake include trace metals [40] and organic contaminants such as organochlorinated pesticides (OCPs), polychlorinated biphenyls (PCBs) and polybrominated biphenyl ethers (PBDEs) [1, 41, 42], which pose potential threats to resident aquatic organisms.

In this study, caged snails were employed to assess sediment quality in the eastern part of Taihu Lake. The objectives were (1) to determine the suitability of a battery of biochemical biomarkers analyzed in caged *B. aeruginosa* as a tool to assess exposure to contaminated sediments under field conditions; and (2) to evaluate the sediment quality of Taihu Lake using a combination of chemical analysis and in vivo toxicity assessments. Specifically, the battery of biomarkers utilized by this study consisted of activities of EROD, antioxidant enzymes including superoxide dismutase (SOD) and catalase (CAT), reactive oxygen species (ROS), the protein carbonyl content (PCC) and lipid peroxidation (LPO). Finally, levels of trace metals, OCPs, PCBs and PBDEs were investigated in sediments of the lake.

## Methods

### Chemicals and reagents

Reference standards of OCPs, including 2,4′-dichlorodiphenyldichloroethylene (2,4′-DDE), 2,4′-dichlorodiphenyldichloroethane (2,4′-DDD), 4,4´-dichlorodiphenyltrichloroethane (4,4′-DDT), 4,4′-dichlorodiphenyldichloroethylene (4,4′-DDE), 4,4′-dichlorodiphenyldichloroethane (4,4′-DDD), alfa-hexachlorocyclohexane (α-H-CH), beta-hexachlorocyclohexane (β-HCH), gamma-hexachlorocyclohexane (γ-H-CH), delta-hexachlorocyclohexane (δ-HCH), aldrin, hexachlorobenzene (HCB), pentachloroaniline (PCA), dieldrin, α-endosulfan, β-endosulfan, endrin, heptachlor, *cis*-heptachlor epoxide, *trans*-heptachlor epoxide, methoxychlor and mirex were purchased as a mixture from AccuStandard (New Haven, USA).

The PCB congeners including CB-18, 28, 31, 44, 52, 101, 118, 138, 149, 153, 170, 180, 194 and 209 were also purchased from AccuStandard (New Haven, USA). PBDE congeners: BDE-28, 47, 66, 99, 100, 153, 154 and 183 were bought as a mixture from Wellington Laboratories Inc. (Guelph, Ontario, Canada). A mixture with octa- to deca-BDEs (BDE-194, 195, 196,197, 198, 199, 200, 201, 202, 203, 204, 205, 206, 207, 208 and 209) was also bought from Wellington Laboratories Inc. (Guelph, Ontario, Canada). Methoxylated polybrominated diphenyl ethers (MeO-PBDEs) congeners, including 2-MeO-BDE68 and 2-MeO-BDE47, were purchased from AccuStandard (New Haven, USA). All solvents used for sample processing and analyses were of pesticide grade.

### Test species

Adults of *B. aeruginosa* were obtained from clean sediments of artificial ponds in Wuhan Botanic Garden (Wuhan, China) and selection of the correct species was confirmed taxonomically [43]. Before initiation of the caging experiments, the snails were acclimatized for more than 3 months in clean glass tanks in the laboratory. During the acclimation, the culture conditions were set based on natural habitat conditions. The ratio of sediments to water was 1:4 and the photoperiod was set at a 12 h light and 12 h dark cycle. The snails were kept in tanks at a water temperature of 24 ± 1 °C, a hardness of 250 mg/L, a pH value of 7.5 ± 0.5 and a dissolved oxygen concentration of 10.5 ± 0.5 mg/L. The water was charcoal filtered and dechlorinated tap water. The sediments were prepared with unpolluted terrestrial soil, which were collected from an uncultivated plot at Dehang Nature Reserve, Jishou, China [22]. The concentrations of trace metals and organic pollutants in sediments are listed in Additional file 1: Table S1. *B. aeruginosa* were fed with commercial aquarium fish food (Sanyuan^®^, Beijing, China) [22]. After acclimation, a group of snails (*n* = 120) was sampled for the verification of baseline levels of biomarkers for this species, and as no significant difference was detected among baseline levels determined for this group. This group was named basal group (Control). 720 snails were transported to the selected experimental sites in plastic bags containing water and oxygen for the caging exposure tests. Snails with an average shell length of 20.00 ± 1.68 mm and average body weight of 2.05 ± 0.20 g were used in the experiments. The experiments were conducted in accordance with the Animal Ethics Committee at Tongji University.

### Study sites

**Fig. 1:**
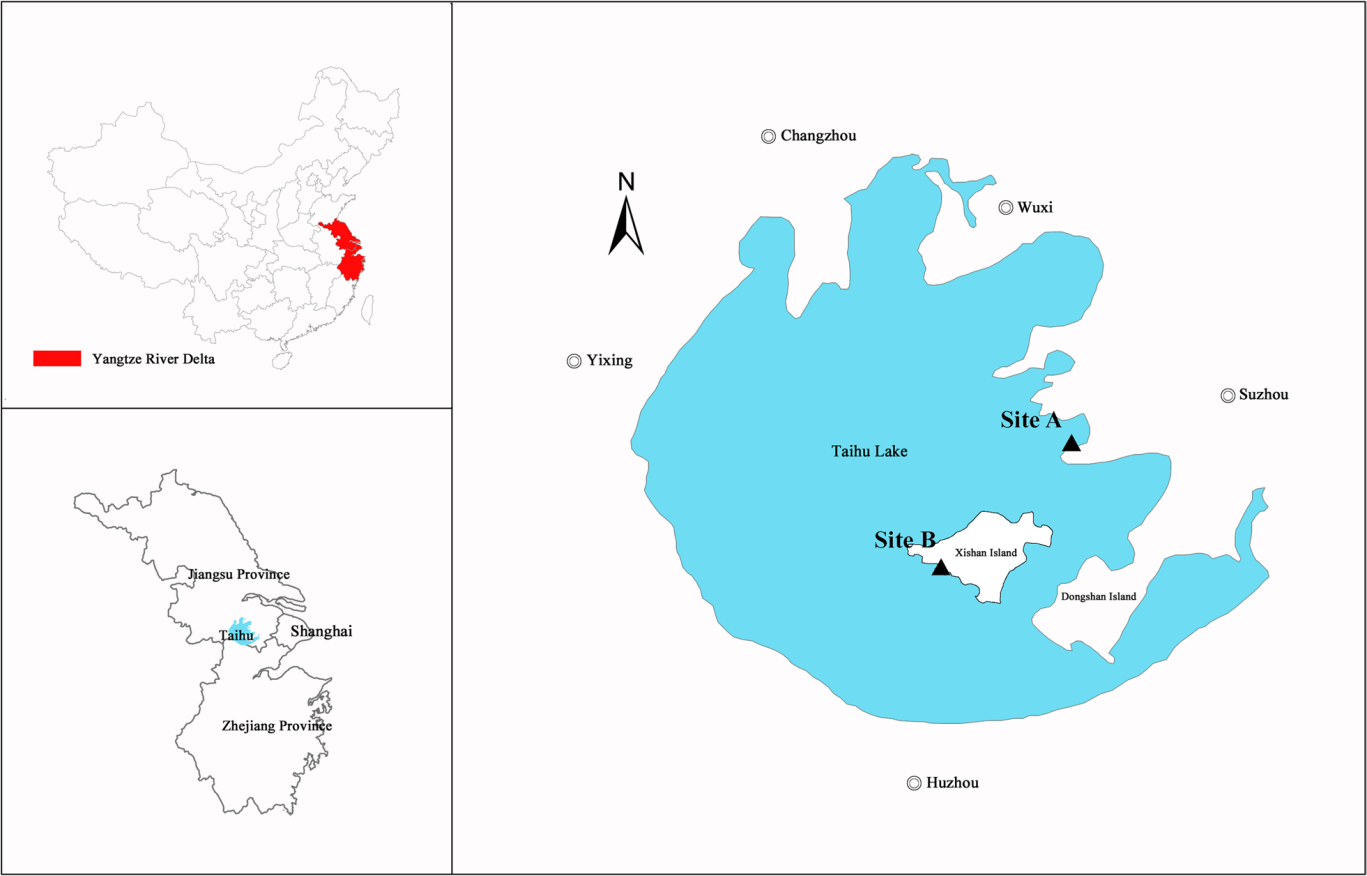
Location of the Yangtze River Delta in China and caging sites in Taihu Lake (solid triangles represent caging sites A and B; double circles around the lake are major cities with population over one million)

In situ exposure tests were performed at two experimental sites in Taihu Lake, site A (31°13′51′′N–120°22′7′′E) and site B (31°6′2′′N–120°13′55′′E) (Fig. 1). Site A was located in the eastern area of Taihu Lake in proximity to the water quality and cyanobacterial field multi-observation station for Taihu Lake in Jiangsu province. Pollutants produced by industry and agriculture were mainly from the east of the lake basin, such as Suzhou City. The eastern area is the major outlet of Taihu Lake and is mostly covered with macrophytes which reduce water flow velocity and increase sedimentation rates [44]. Site B was located at Xishan Island in the central lake area. This island has been developed for tourism for several decades, so many vessels for fishery and tourism were built, which have caused pollution in this area. The average water depth of the two sites in Taihu Lake is about 2.0 m. In some previous studies, a reference site was selected in comparatively uncontaminated environment [12, 45]. However, it is scarcely possible to find such a clean freshwater area without anthropogenic pollutants in China, thus the basal group was determined as the control group following other studies [10, 27, 46].

### Caging exposure

Caged snail exposure tests were conducted for 21 days during August 2015. Cylindrical cages (0.26 m height, 0.80 m diameter, 130 L volume) composed of nylon mesh (3 mm) and a polypropylene baffle were used for the exposures. At each site, three cages, containing 120 snails each, were immersed into the water and fixed in direct contact with the sediments. One cage from each site was sampled each after 7, 14 and 21 days, respectively, and surviving organisms were counted. A subset of snails was immediately frozen in liquid nitrogen for biochemical analysis. However, one cage located at site A disappeared unexpectedly for unknown reasons after 21 day of exposure, and thus it was impossible to recover the snails from this cage. The caged snail samples exposed for 7 and 14 days, and 7, 14 and 21 days at sites A and B were defined as *A*_7_ and *A*_14_, and *B*_7_, *B*_14_ and *B*_21_, respectively. Water quality parameters, including temperature, pH and dissolved oxygen, were measured during the caging exposure by the Jiangsu water quality and cyanobacteria field multi-observation station for Taihu Lake.

### Sediment sampling and chemical analysis

Surface sediment samples (0 to 5 cm depth) were collected from each caging site at the beginning of the encaged experiment using a Van Veen grab and were brought to the laboratory immediately. Sediments were freeze-dried, homogenized, ground and sieved with a 200-mesh nylon screen and kept below − 20 °C until chemical analysis.

#### TOC content

Total organic carbon (TOC) contents of the sieved and freeze-dried sediments were determined using a total organic carbon analyzer (TOC-VCPN, Shimadzu, Japan).

#### Trace elements

The sieved and freeze-dried samples (0.2 g) were microwave digested in Teflon vessels using a mixture of concentrated HNO_3_, HCl and HF according to the EPA Method 3052 [47]. HClO_4_ was added to remove HF and then adjusted to a volume of 10.0 mL with 2% (v/v) HNO_3_ before instrumental analysis [48]. Target elements (Cr, Cu, Pb, Ni, Zn, Cd, As) were analyzed using inductively coupled plasma atomic emission spectroscopy (ICP-AES, Agilent 720 ES, USA). Recovery rates of the analytical procedures were assessed using a standard reference material (SRM 1646a, National Institute of Standards and Technology), and all elements showed a recovery greater than 90% of the certified concentration.

#### Organic pollutants

The procedures for the extraction and purification of organochlorine pesticides (OCPs), polychlorinated biphenyls (PCBs) and polybrominated diphenyl ethers (PBDEs) have been published in detail elsewhere [49–52]. So the methods for extraction, clean up, analysis and quality assurance and quality control are described in Additional file 1: Text S1.

### Biochemical markers

Thirty snails from each cage were dissected and their hepatopancreas was removed for biomarker determination. Hepatopancreas tissue was weighed and homogenized (1:4 w/v) in ice-cold Tris–HCl buffer (0.01 M, pH 7.4) and 1.0 mM phenylmethylsulfonyl fluoride as a serine protease inhibitor using a glass homogenizer. The homogenates were centrifuged at 1000×*g* for 10 min at 4 °C and the section of supernatants was collected for determination of protein carbonyl content. The remaining content was continuously centrifuged at 9000×*g* for 20 min at 4 °C and the resulting supernatant was separated for the analysis of other biochemical parameters. Total protein concentrations were determined with the total protein kit (Sigma-Aldrich, USA) based on the Bradford method [53]. All biomarker responses were detected using BioTek Synergy 4 multi-mode microplate reader (BioTek, USA).

#### Ethoxyresorufin-*O*-deethylase (EROD) activity

EROD activity of the supernatant (S9 fraction) was measured following the hydroxylation of 7-ethoxyresorufin according to the method of Burke and Mayer [54] as adapted by Pacheco and Santos [55]. The reaction mixture consists of 950.0 μL phosphate-buffered saline (PBS, pH 7.8; Sigma, USA), 10.0 μL of 0.1 mM ethoxyresorufin (ERF; Sigma-Aldrich, USA), 30.0 μL of 1.0 mM NADPH (Aladdin, USA) and 10.0 μL prepared hepatopancreas homogenate supernatant sample. Determination of enzyme activity of each sample was carried out by measuring the end product resorufin (RF) of the catalytic reaction using the fluorescence multi-well plate reader (BioTek, USA) at excitation wavelength 530 nm and emission wavelength 585 nm. Enzyme activity was calculated as pmol/min/mg protein using a standard curve of resorufin.

#### Reactive oxygen species (ROS) production

ROS (superoxide and hydroxyl radicals) were detected with the Fluorometric Intracellular ROS Assay Kit (Sigma-Aldrich, USA) following the manufacturer’s protocol using fluorometric sensors localized to the cytoplasm, resulting in a fluorometric product proportional to the amount of ROS present. The fluorescence intensity was determined at λex = 540 nm and λem = 570 nm. Fold increases in ROS levels were determined by comparison with the control group.

#### Antioxidant enzymes

SOD activity was determined by the SOD assay kit (Sigma-Aldrich, USA). SOD activity was quantified by measuring the decrease in the color development at 440 nm with a colorimetric method, which is proportional to the amount of superoxide anion. Results were expressed as the inhibition rate (%) of the reduction with molecular oxygen.

Changes in CAT activity were evaluated by the catalase assay kit (Sigma-Aldrich, USA). CAT activity was quantified by measuring the hydrogen peroxide substrate remaining after reaction for 15 min by an absorbance spectrophotometer at 520 nm. Results were expressed as mmole/min/mL.

#### Oxidative stress

LPO was determined by the lipid peroxidation assay kit (Sigma-Aldrich, USA) by measuring the colorimetric product (532 nm) formed by the reaction of malondialdehyde (MDA) with thiobarbituric acid (TBA) and which is proportional to the concentration of MDA present. Results were expressed as nmole/μL.

PCC assay kit (Sigma-Aldrich, USA) was applied to detect the production of stable carbonyl groups induced by oxidative stress. Carbonyl content was determined by the derivatization of protein carbonyl groups with 2,4-dinitrophenylhydrazine (DNPH) leading to the formation of stable dinitrophenyl (DNP) hydrazone adducts, which can be detected spectrophotometrically at 375 nm, proportional to the carbonyls present. Results were expressed as nmole/mg protein.

### Integrated biomarker response index (IBR)

The biomarker results were applied into the “Integrated Biomarker Response Index” (IBRv2) described by Beliaeff and Burgeot [32] and modified by Sanchez et al. [35]. This novel version of IBR is based on the principle of reference deviation between a disturbed and undisturbed state [35]. In the present study, the deviation between biomarkers measured in snails caged at site A and site B were compared to those measured in snails acclimated in the sediments under controlled laboratory conditions (baseline levels). The baseline values of each biomarker (*X*_0_) were used as a reference value.

For each individual biomarker, the ratio between the mean value (*X*_*i*_) obtained at each caging site and the respective mean baseline (*X*_0_) was log-transformed (*Y*_*i*_) (Eq. 1):

1 $$Y_{i} = { \log }\left( {X_{i} /X_{\text{0}} } \right).$$

In the next step, the general mean (*μ*) and standard deviation (*s*) of *Y*_*i*_ were computed. Then, *Y*_*i*_ values were standardized by the formula (Eq. 2):

2 $$Z_{i} = \, \left( {Y_{i} - \mu } \right)/s.$$

The differences between the mean of standardized biomarker response (*Z*_*i*_) and mean of reference biomarker data (*Z*_0_) were used to define the biomarker deviation index (*A*) (Eq. 3):

3 $$A = Z_{i} - Z_{0} .$$

Finally, the integrated multiple biomarkers response index (IBR) of the caged snails in each caging site was obtained by summarizing the absolute value of *A* parameters for each biomarker.

### Statistical analysis

Statistical analysis of data was carried out by SPSS 19.0. Biomarker results were tested for normality and homogeneity of variance with the Shapiro–Wilks and Levene test, respectively. Analysis of variance (ANOVA) followed by a Dunnett’s test was used to compare the biomarkers obtained in snails from the two caging sites after different exposure times with those obtained in snails acclimated in the laboratory. The significance level was set at *p *< 0.05.

## Results

### Physical and chemical analysis of water and sediments

There were no differences in water quality between the two sampling sites, with water temperatures of 26.8–33.1 °C, pH of 8.2–8.7 and dissolved oxygen (DO) concentrations of 6.57–9.16 mg/L.

Chemical characterization of the sediment samples from the caging sites identified 7 trace elements and 55 organic compounds (21 OCPs, 11 PCBs and 23 PBDEs) (Table 1). With the exception of PBDEs, concentrations of metals and other organic pollutants were markedly greater at site A when compared to site B.

**Table 1:** Chemical analysis of surface sediments collected from the caging sites A and B in the Taihu Lake

Parameters		Site A	Site B	TEL^a^	PEL^b^
TOC (%)		0.77	1.29		
Metals (mg/kg)	Cr	34.17	28.99	37.30	90.00
	Cu	39.49	17.72	35.70	197.00
	Pb	24.15	23.85	35.00	91.30
	Ni	21.40	16.89	18.00	36.00
	Zn	99.00	62.47	123.00	315.00
	Cd	0.20	0.16	0.60	3.53
	As	6.77	5.04	5.90	17.00
OCPs-DDTs (ng/g dw)	2,4′-DDD	0.36	0.01		
	2,4′-DDE	*0.001*	0.13		
	4,4′-DDD	*0.001*	0.04	3.54	8.51
	4,4′-DDE	3.45	1.68	1.42	6.75
	4,4′-DDT	0.45	0.13		
OCPs-HCHs (ng/g dw)	α-HCH	*0.001*	0.01		
	β-HCH	0.54	0.01		
	γ-HCH	0.64	0.04	0.94	1.38
	δ-HCH	32.03	*0.001*		
Other OCPs (ng/g dw)	Aldrin	*0.001*	*0.001*		
	HCB	*0.001*	0.06		
	PCA	0.57	0.01		
	Diedrin	1.15	0.01		
	α-Endosulfan	*0.001*	0.01		
	β-Endosulfan	*0.001*	*0.001*		
	Endrin	*0.001*	0.01	2.67	62.4
	Heptachlor	*0.001*	*0.001*		
	*cis*-heptachlor epoxide	*0.001*	0.01		
	*trans*-heptachlor epoxide	*0.001*	0.02		
	Methoxychlor	1.32	*0.001*		
	Mirex	*0.001*	0.05		
PCBs (ng/g dw)	CB18	3.43	0.13		
	CB31 + 28	3.10	0.02		
	CB44	9.31	0.02		
	CB52	*0.001*	0.03		
	CB101	*0.001*	*0.001*		
	CB138	0.21	0.05		
	CB153	0.84	*0.001*		
	CB180	0.54	0.05		
	CB183	*0.001*	0.03		
	CB194	0.33	0.02		
	CB209	*0.001*	*0.001*		
PBDEs (ng/g dw)	BDE85	*0.001*	*0.001*		
	BDE154	*0.000*	0.01		
	BDE153	0.01	0.01		
	BDE183	0.02	0.01		
	BDE202	*0.001*	*0.001*		
	BDE201	0.02	0.01		
	BDE204	*0.001*	*0.001*		
	BDE197	*0.001*	0.01		
	BDE (198 + 199 + 200 + 203)	*0.000*	*0.000*		
	BDE196	0.03	0.01		
	BDE205	*0.000*	0.01		
	BDE194	0.02	0.04		
	BDE195	*0.001*	0.15		
	BDE208	0.07	0.02		
	BDE207	*0.001*	0.02		
	BDE206	0.14	0.03		
	BDE209	0.35	0.36		
	BDE47	*0.001*	0.01		
	BDE66	0.01	*0.001*		
	2-MeO-BDE68	*0.001*	*0.001*		
	2-MeO-BDE47	*0.001*	*0.001*		
	BDE100	*0.001*	*0.001*		
	BDE99	*0.001*	0.01		
ΣDDTs (ng/g dw)		4.26	1.99	7.00	4450.00
ΣOCPs (ng/g dw)		40.50	2.22		
ΣPCBs (ng/g dw)		17.76	0.35	34.1	277
ΣPBDEs (ng/g dw)		0.67	0.71		

Detected concentrations are presented in plain type. Values in italic type mean not detected and are presented by limit of detection (LOD). *Dw* dry weight

^a^ Threshold effect level (Smith et al. [56])

^b^ Probable effect level (Smith et al. [56])

### Individual mortality

**Fig. 2:**
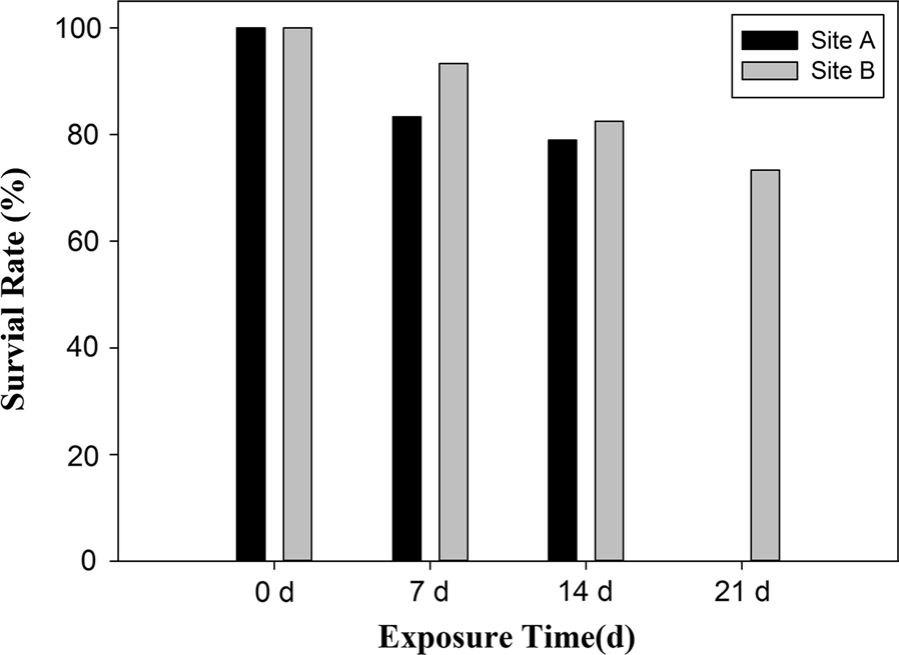
Survival rate (%) of *Bellamya aeruginosa* caged at sites A and B during the caging exposure in Taihu Lake

The survival rates decreased with the increasing exposure time at both sites (Fig. 2). The survival rate of caged snails at site A decreased to 78.9% after exposure for 14 days, which was slightly lower than the survival rate of snails caged at site B (82.5%). The survival rate for site B was reduced to 73.3% after 21 days of exposure.

### Biomarkers

#### Detoxification metabolism

**Fig. 3:**
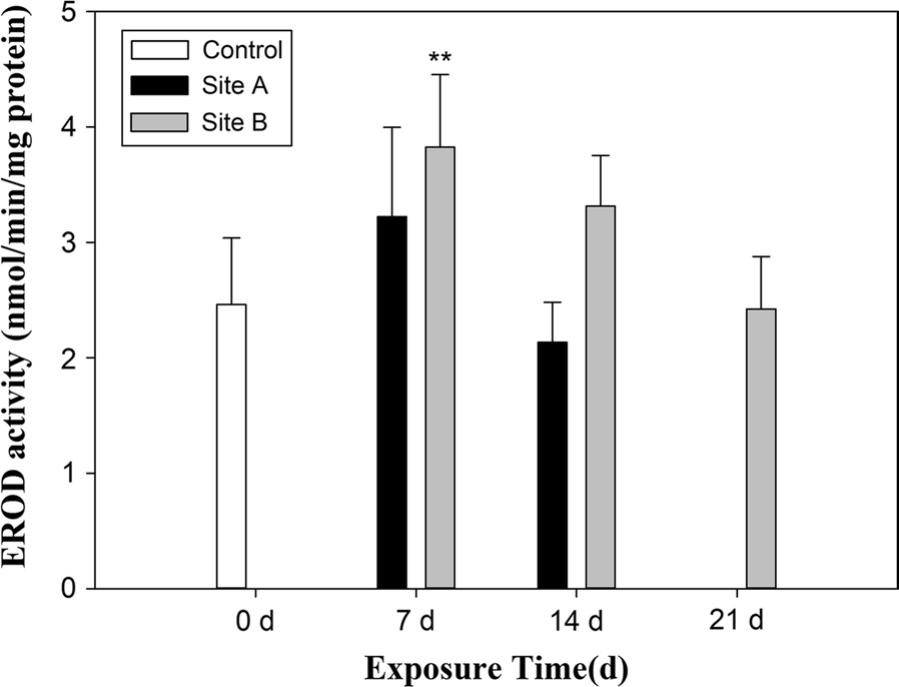
EROD activities in the hepatopancreas of snails (*Bellamya aeruginosa*) caged at sites A and B over the duration of exposure experiments in Taihu Lake. Results are mean ± SD. Data represent the average of 30 individuals in each sample. Double asterisks indicate significant difference from the control (*P *< 0.01)

With regard to EROD activity, there was a significant increase only in snails caged at the site B after 7 days (*p* < 0.01), while no significant differences were observed between snails confined at the site A compared with the control group (Fig. 3).

#### Reactive oxygen species

**Fig. 4:**
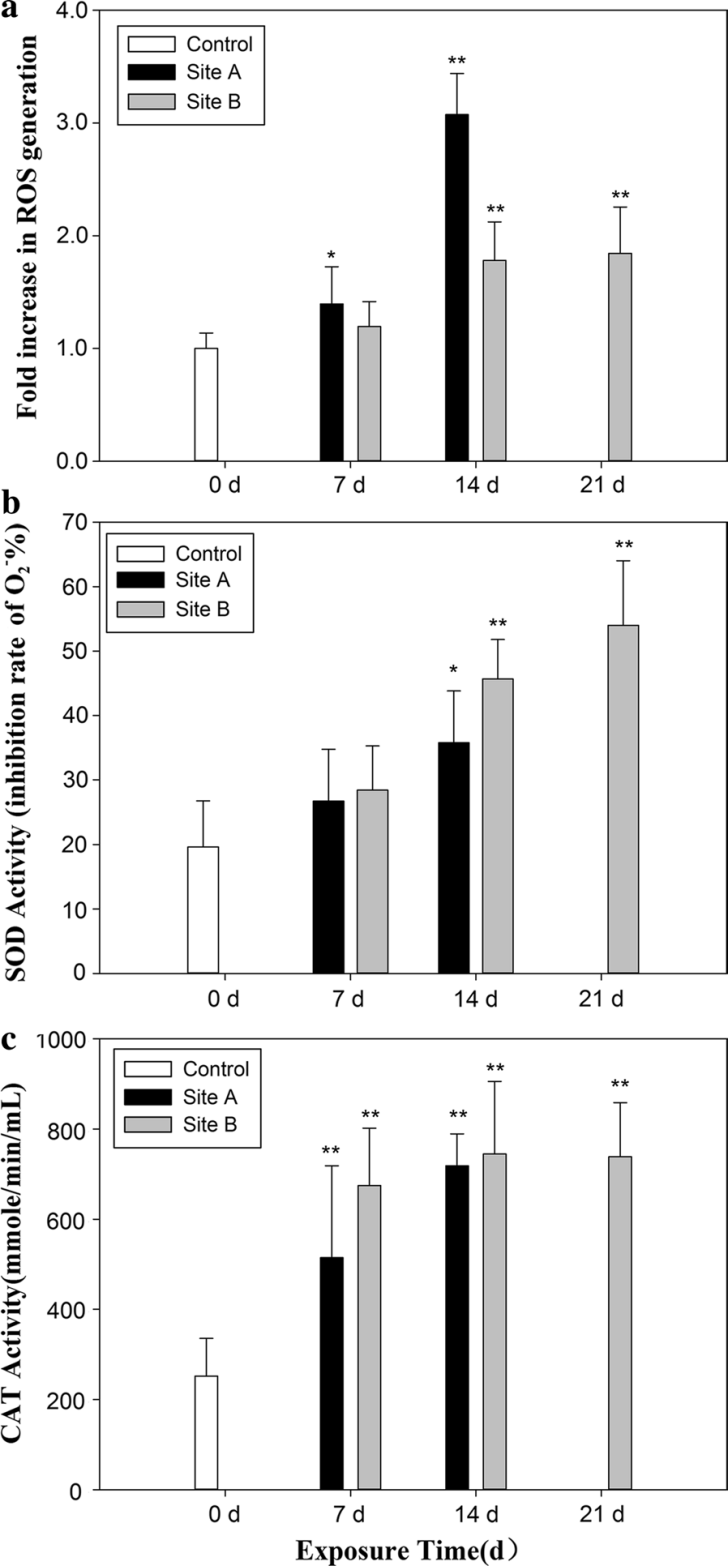
Fold increase in ROS generation compared to the control (**a**) and activities of antioxidant enzymes (SOD and CAT activities) (**b**, **c**) in the hepatopancreas of snails (*Bellamya aeruginosa*) caged at sites A and B over the duration of the exposure experiments in Taihu Lake. Results are mean ± SD. Data are given from 30 individuals in each sample. Asterisks indicate significant difference from the control (*P *< 0.05); double asterisks indicate significant difference from the control (*P *< 0.01)

The levels of ROS in the hepatopancreas of caged snails at both sites A and B showed a time-dependent and significant increase compared to snails from the control group (Fig. 4a). Specifically, ROS levels in snails from site A increased to approximately three times that of those measured in the controls after 14 days of exposure. Maximum increases of ROS in snails from site B were 1.8-fold relative to controls and were significantly less than the levels in animals exposed at site A (*p *< 0.05).

#### Antioxidant defenses

Significant and time-dependent increases in activities of antioxidant enzymes were observed in snails exposed at both sampling sites (Fig. 4b, c). SOD activities increased linearly with exposure time at sites A (*p *< 0.05; Fig. 4b) and B (*p *< 0.01; Fig. 4b). CAT activities of caged snails at both sites A and B were also significantly increased at all exposure times compared with the control group (*p *< 0.01; Fig. 4c).

#### Oxidative stress

**Fig. 5:**
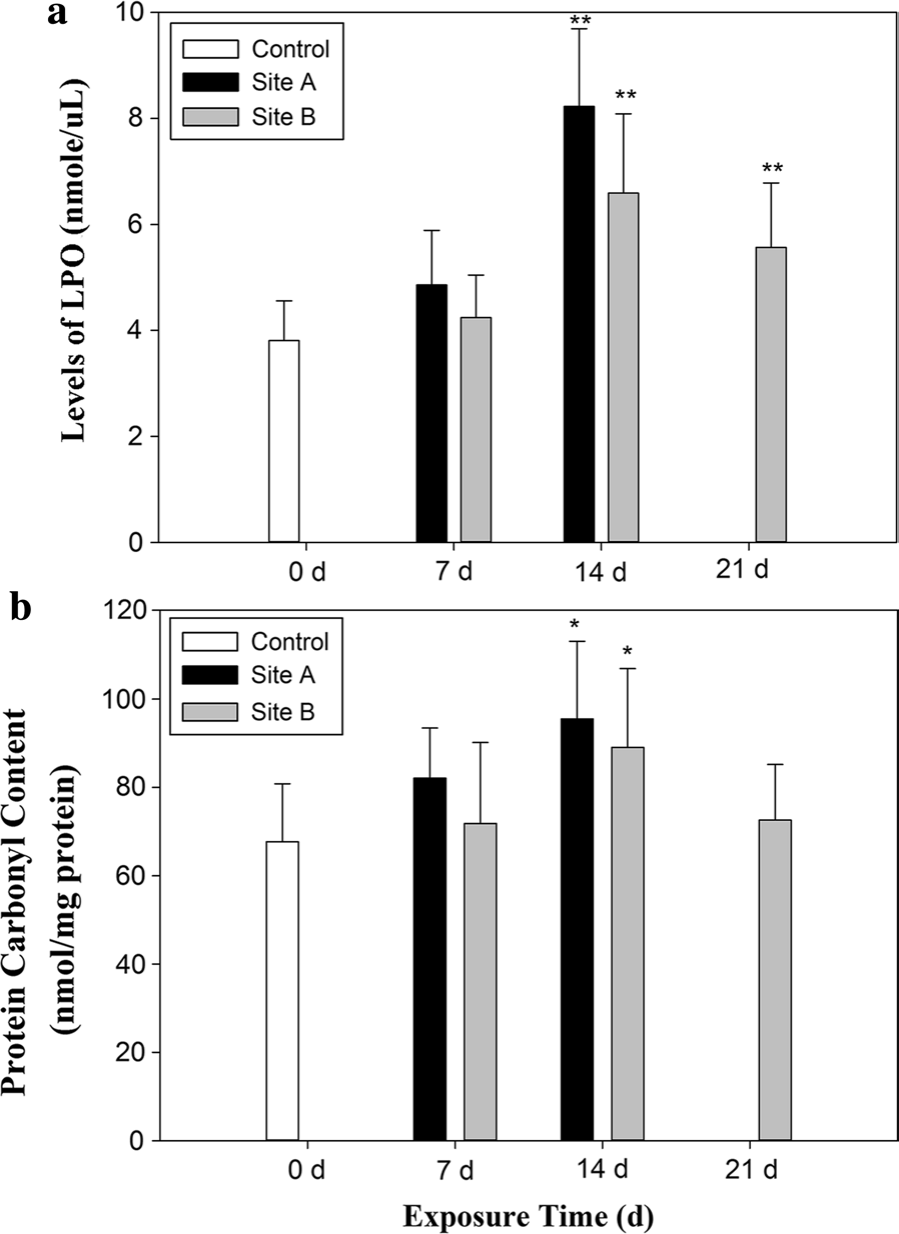
Oxidative effects [the levels of LPO (**a**), PCC (**b**)] in the hepatopancreas of snails (*Bellamya aeruginosa*) caged at sites A and B over the duration of the exposure experiments in Taihu Lake. Results are mean ± SD. Data are given from 30 individuals in each sample. Asterisks indicate significant difference from the control (*P *< 0.05); double asterisks indicate significant difference from the control (*P *< 0.01)

During the initial 14 days of exposure, levels of LPO and PCC in the hepatopancreas of caged snails at both sites increased with the increasing exposure time (Fig. 5). Significant increases in LPO and PCC levels were observed after exposure for 14 days at both sites compared to the control group (*p *< 0.01 and *p *< 0.05, respectively). Then the levels of LPO and PCC decreased on day 21 at site B. While LPO was still significantly higher in snails from site B than the control group (*p *< 0.01), no such difference was observed for the levels of PCC in the hepatopancreas of caged snails after 21 days at this site (*p *< 0.05).

### Integrated biomarker response index

**Fig. 6:**
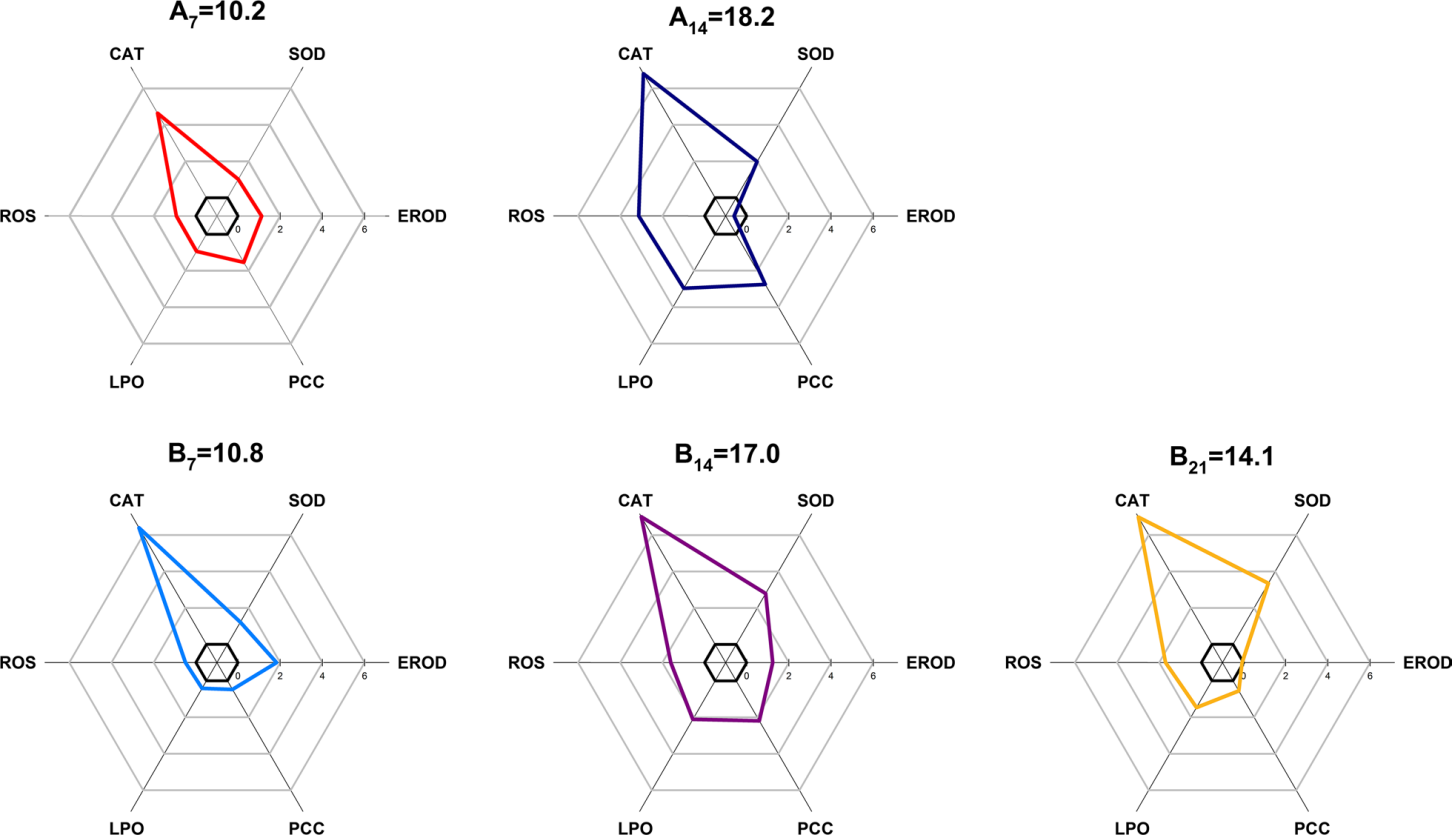
Integrated biomarker responses index (IBRv2) based on the following biomarkers: ethoxyresorufin-*O*-deethylase activity (EROD), superoxide dismutase (SOD), reactive oxygen species (ROS), lipid peroxidation (LPO), the protein carbonyl content (PCC) and catalase activity (CAT) in the hepatopancreas tissue of *Bellamya aeruginosa* caged at the site A and site B of the Taihu Lake. For both sites A and B, *A* values calculated for each biomarker were reported in a star plot indicating the reference deviation of each investigated biomarker. Biomarker results represented in relation to the baseline group. The area above 0 reflects induction of biomarker and below 0 indicates reduction of the biomarker

All biochemical biomarkers showed a significant induction in the hepatopancreas of snails as a function of exposure time, with the exception of EROD activity at site A (Fig. 6). The biomarker deviation index (*A*) of CAT activity was greater than that of all other biomarkers, which indicated that CAT was the most responsive biomarker in this in situ exposure experiment. The IBR values for both sites A and B were 10.2 and 10.8 after caging for 7 days, respectively, as minor variations in the analyzed biomarkers were observed. After exposure for 14 days, both caging sites A and B showed the highest IBR value (*A*_14_ = 18.2, *B*_14_ = 17.0). The IBR value for site B decreased to 14.1 after 21 days of exposure.

## Discussion

Biomarkers are important tools for understanding linkages between external contaminant exposures, internal doses and associated potential impacts on health status [12]. In this study, sediment quality of Taihu Lake was assessed by using snails *B. aeruginosa* caged in situ, integrating multiple biochemical responses and chemical analysis.

Analyses of selected contaminants in sediments of Taihu Lake showed that metals (Cr, Cu, Pb, Ni, Zn, Cd, As), OCPs, PCBs and PBDEs were detectable at both caging sites. Sediment quality assessment values for freshwater ecosystems in Canada [56] were consulted to evaluate potential risks associated with the observed chemicals. These guidelines consider two effect levels for the characterization of potential toxicological risks, the threshold effect level (TEL) and the probable effect level (PEL). The TEL represents the concentrations of these contaminants below which adverse biological effects only rarely occur, while the PEL represents the concentrations of these contaminants above which adverse biological effects frequently occur [56]. In this study, none of the chemical contaminants detected in sediments from both sites were found to exceed the PEL threshold values (Table 1). However, concentrations of Cu, Ni and As in sediments from site A were greater than their TEL. For OCPs, the concentrations of 4,4′-DDE in sediments from site A and site B were found to be above the TEL. Similarly, Zhao et al. [41] demonstrated that high residues of OCPs, including DDTs, HCHs, heptachlors and chlordanes, persisted in surface sediments and *B. aeruginosa* collected at 20 locations in Taihu Lake. Thus, there is the potential for chemical compounds in sediments from Taihu Lake to have adverse effects on aquatic organisms. In addition, all concentrations of chemicals analyzed in the sediment collected from site A, except for PBDEs, were greater than those at site B. This is in accordance with the result that the survival rate of *B. aeruginosa* caged at site A was lower than that of snails caged at site B after different exposure times (7 and 14 days), indicating that *B. aeruginosa* was a relatively sensitive species for biomonitoring in Taihu Lake.

Xenobiotics, especially lipophilic pollutants, can induce adaptive changes in exposed organisms such as induction of phase I enzymes that can oxidize xenobiotics to make them more polar, and thus allow them to be conjugated and excreted. In this study, the mixture of contaminants in sediments of Taihu Lake significantly induced EROD activity in hepatopancreas tissues of snails from both sites after exposure for 7 days. Dioxin-like contaminants, including some PCBs [57], certain polycyclic aromatic hydrocarbons (PAHs) [58] and polychlorinated dibenzo-*p*-dioxins and dibenzofurans (PCDD/Fs) [12] have been reported to activate the aryl hydorcarbon receptor, which results in the induction of EROD activity. Dickerson et al. [59] also demonstrated that 4′4-DDE could cause a dose-dependent increase in EROD activity in the deer mouse. After exposure for 14 days, there was no significant difference in the EROD activity between the caged snails at both sites and the control, which indicated an adaptation of the caged snails for the contaminated sediments at both sites in Taihu Lake.

Pollutants such as PCBs, lindane and certain heavy metals are important triggers of oxidative stress and the formation of ROS in biological systems [60, 61]. In this study, increased ROS levels were observed in hepatopancreas of caged snails at both sites (Fig. 4), indicating exposure to compounds that cause oxidative stress. Antioxidant enzymes can be induced by enhanced production of ROS as a protective mechanism against oxidative stress, or they can be inhibited when deficiency of the system occurs, suggesting toxicity [62]. In this study, significant increase in oxidative stress as measured by ROS concentrations as well as LPO and PCC occurred in snails exposed at both sampling locations. In parallel, significant increases in SOD and CAT activities were observed, which suggested an adaptive response of the snails inhabiting contaminated sites in Taihu Lake. One possible reason for this increase could be the elevated concentrations of metals including Cu, Ni and As, which exceeded TEL values. In an earlier study, Ma et al. [22] found that hepatopancreatic SOD and CAT activities in *B. aeruginosa* were significantly increased after exposure to 51 mg/kg of Cu for 28 days. These concentrations were similar to those measured during our study (Cu concentrations ranged between 17.7 and 39.5 mg/kg). Thus, together with the other elements measured in sediments from Taihu Lake, we hypothesize that metals were likely to have caused the oxidative stress observed in caged snails during the exposure.

Lipid peroxidation is the process of oxidative degradation of lipids, which is regarded as a measure of oxidative stress induced by environmental pollutants including heavy metals and wide ranges of organic pollutants [63, 64]. In addition, proteins can be attacked by ROS, which will lead to the formation of carbonyl, a process that is non-reversible and causes a conformational change of proteins [65]. Such changes will reduce catalytic activity in enzymes and ultimately result in breakdown of proteins [66]. In this study, the levels of LPO and PCC in the hepatopancreas of caged snails at both sites significantly increased after exposure for 14 days compared to the control group. The temporal variation of LPO and PCC levels indicated that oxidative stress was induced by contaminants in sediments. LPO and PCC levels in the hepatopancreas of the caged snails at site B were ameliorated at the later time point of 21 days compared with 14 days, which might be partly due to the increased biochemical defense responses (SOD and CAT) observed in snails at this site. The decrease in both LPO and PCC levels on day 21 indicated an adaptation of the caged snails to the exposure to the environmental conditions in Taihu Lake.

Variations in biochemical biomarkers depend on food availability and spawning period besides exposure to pollutants [67]. Preliminary experiments showed that the inter-individual variations of measured biomarker responses in the basal group were relatively low. As there was enough organic matter in the sediments of Taihu Lake, *B. aeruginosa* could get enough food during the caging experiment [68]. Xiong et al. [69] have also demonstrated that the CAT activities of *B. aeruginosa* increased in Suzhou Creek for 0, 15 and 30 days and did not show significant difference (*p* < 0.05). Abiotic factors such as temperature, salinity and dissolved oxygen content also influence biomarker responses in an organism [70]. The analysis of water quality revealed no variations between the two caging sites, with values within the normal range for this environment. So abiotic factors may have the same influence on snails caged in two sites. However, possible variations in some specific biomarker responses could not be avoided in the field study [13]. So the combination of biomarkers could give a comprehensive picture and provides better insights to study the effects of pollutants.

The Integrated Biomarker Response Index (IBRv2) has been found capable of discriminating scores to describe toxically induced stress based on the specific responses of a number of biomarkers across different sampling sites [27]. In this study, it was used as a tool for the visualization of biological effects of contaminants on snails caged at both sites in Taihu Lake. Several biomarkers exhibited a response that was induced or inhibited as a function of sampling site, and spatial arrangement of these biomarkers in the radar chart allowed visualizing the most sensitive biomarkers in the evaluation [71]. From the radar chart of IBR (Fig. 6), CAT activity was the most responsive biomarker compared with other multiple biomarkers in this study. CAT is an important enzyme in antioxidant defense systems protecting animals from oxidative stress [10, 33] and has been studied in *B. aeruginosa* [22] and land snails (*Theba pisana*) [72]. In general, the IBR index (stress index) increased from day 7 to day 14 of the exposure and decreased at day 21, showing that environmental stress effects in *B. aeruginosa* initially increased and then weakened, which might be an adaptation mechanism for *B. aeruginosa* to toxic effects of environmental contaminants. The IBR values were higher in animals from site A on the 14th day of exposure compared to site B, indicating that site A had a greater impact on *B. aeruginosa*. As higher contamination levels of metals, OCPs and PCBs were found in the sediments collected from site A compared to site B, the IBR index proved to be in agreement with the levels of environmental contamination in different sediments from Taihu Lake. Thus, it could be inferred that the integration of multiple biomarker responses found in caged snails could reflect the contamination levels measured at different sites and used as an efficient biomonitoring approach [27, 34]. However, comparisons among larger numbers of diverse sites are required to further validate the IBR as a predictive tool to categorize the contamination levels of sediments.

## Conclusion

The biochemical responses in caged snails deployed at two sites in Taihu Lake as well as the chemical analysis of selected organic and inorganic pollutants indicated significant anthropogenic pollution and potential risks to resident organisms. Multiple biomarkers including EROD, ROS, SOD, CAT, LPO and PCC were induced during caging exposure experiment. The IBRv2 results showed that the CAT activity was the most responsive biomarker. The results of the biochemical biomarkers in *B. aeruginosa* were consistent with the chemical contamination in the sediments of the lake, indicating that in situ exposures with caged snails can serve as an efficient biomonitoring approach to evaluate sediment quality.

## Additional file

**Additional file 1: Table S1.** Chemical analysis of surrogate sediments used in the laboratory culture of *Bellamya aeruginosa.* **Text S1.** Organic pollutants.
