# Supplementary material for: Multiple biomarker responses in caged benthic gastropods Bellamya aeruginosa after in situ exposure to Taihu Lake in China
Source: Environ Sci Eur. 2018 Sep 11;30(1):34. doi: 10.1186/s12302-018-0164-y (PMC6132844; doi:10.1186/s12302-018-0164-y)
Supplement: Supplementary file 1 — Additional file 1: Table S1. Chemical analysis of surrogate sediments used in the laboratory culture of Bellamya aeruginosa. Text S1. Organic pollutants. [file 12302_2018_164_MOESM1_ESM.docx]

**Additional file**

Multiple biomarker responses in caged benthic gastropods *Bellamya aeruginosa* after *in situ* exposure to Taihu Lake in China

Qian Li^1^, Meng Wang^1^, Lei Duan^1^, Yanling Qiu^1^, Taowu Ma^2^, Ling Chen^1^, Magnus Breitholtz^3^, Åke Bergman^4^, Jianfu Zhao^1^, Markus Hecker^5^, Lingling Wu^1,5^*

^1^ State Key Laboratory of Pollution Control and Resource Reuse, College of Environmental Science and Engineering, Tongji University, Shanghai 200092, China

^2^ College of Biology and Environmental Science, Jishou University, Jishou 416000, China

^3^ Department of Environmental Science and Analytical Chemistry, Stockholm University, Svante Arrhenius väg 8, SE-11418 Stockholm, Sweden

^4^ Swedish Toxicology Sciences Research Center (Swetox), Forskargatan 20, Södertälje 15136, Sweden

^5^ School of the Environment & Sustainability and Toxicology Centre, University of Saskatchewan, 44 Campus Drive, Saskatoon SK S7N 5B3, Canada

*Corresponding Author

E-mail: wulingling@tongji.edu.cn

Tel: +86-21-65984261

**The SI contains 5 pages:**

**Texts:** S1. Organic pollutants

**Tables:** S1. Chemical analysis of surrogate sediments used in the laboratory culture of *Bellamya aeruginosa*

**Text S1 Organic pollutants**

Briefly, the sieved and freeze-dried sediments (10.0 g) spiked with surrogates were Soxhlet extracted with cyclohexane/acetone (1:1, v/v; GC residue analysis, CNW, China) for 48 h. The extracts were then concentrated to a volume of about 1.0 mL in a rotary vacuum evaporator. Isopropanol and saturated sodium sulphite solution were added to remove elemental sulfur. Samples were further cleaned using a mixed silica gel column (from the bottom to top: glass wool, 0.1 g neutral silica gel and 0.9 g sulfuric acid silica gel) and eluted with cyclohexane/dichloromethane (1:1, v/v; GC residue analysis, CNW, China). The extracts were concentrated to 1.0 mL under a gentle N_2_ stream, and re-dissolved in *n*-hexane.

For PBDE analysis, the final volume was reduced to 0.2 mL and a known amount of internal standard BDE118 (2.0 ng; AccuStandard, USA) for PBDEs was added prior to GC-MS (Agilent 6890/5975) analysis in selected ion monitoring mode (bromide isotopes m/z 79 and 81). The injection volume was 1 μL and the temperature for the injector was 280 ºC. A DB-5 column (15m×0.25 mm×0.1μm) from J&W was used. Helium was used as carrier gas at a flow of 1.4 mL/min. The ion source temperature was set to 250 ºC and the transfer line temperature to 290 ºC. Methane was used as reagent gas. The oven temperature program was as follows: initially at 110 °C (held for 1 min) and increased to 200 °C at 20 °C/min (held for 4.5 min), 310 °C at 10 °C/min (held for 5 min). The surrogate (BDE139 (AccuStandard, USA) recoveries for PBDEs were in the range of 72-101%.

For PCB and OCP analysis, concentrated samples were loaded on a silica column and eluted with 25.0 mL of *n*-hexane. The final volume was concentrated to 0.2 mL and a known amount of internal standard CB189 (3.5 ng; AccuStandard, USA) for PCBs and ε-HCH (5.0 ng; Dr. Ehrenstorfer, Germany) for OCPs were added before GC-ECD (Agilent 7890 A) analysis. The splitless-mode injection volume was 1 μL. The temperature was 250 ºC for the injector and 325 ºC for the detector. A DB-5 column (25 m×0.25 mm×0.25 μm) from Agilent Technology was used. Nitrogen was used as carrier gas. The column temperature was initiated at 80 °C (held for 2 min) and increased to 195 °C at 10 °C/min, 230 °C at 3 °C/min, 310 °C at 10 °C/min (held for 5 min). The surrogate (CB53 (AccuStandard, USA) recoveries for PCBs and OCPs were in the range of 70%-97%.

**Table S1. Chemical analysis of surrogate sediments used in the laboratory culture of *Bellamya aeruginosa***

|  |  | Lab |
| --- | --- | --- |
| TOC (%) |  | 4.78 |
| Metals (mg/kg) | Cr | 13.41 |
|  | Cu | 19.74 |
|  | Pb | 18.91 |
|  | Ni | 11.34 |
|  | Zn | 44.98 |
|  | Cd | 0.18 |
|  | As | 3.08 |
| DDTs  (ng/g dw) | 2'4-DDD | ND |
|  | 2'4-DDE | ND |
|  | 4'4-DDD | ND |
|  | 4'4-DDE | ND |
|  | 4'4-DDT | 0.146 |
| HCHs  (ng/g dw) | α-HCH | ND |
|  | β-HCH | ND |
|  | γ-HCH | 0.082 |
|  | δ-HCH | ND |
| Other OCPs (ng/g dw) | Aldrin | ND |
|  | HCB | 0.010 |
|  | PCA | 0.086 |
|  | Diedrin | ND |
|  | α-Endosulfan | ND |
|  | β-Endosulfan | ND |
|  | Endrin | ND |
|  | Heptachlor | 0.081 |
|  | cis-heptachlor epoxide | ND |
|  | trans-heptachlor epoxide | ND |
|  | Methoxychlor | ND |
|  | Mirex | 0.061 |
| PCBs  (ng/g dw) | CB18 | ND |
|  | CB31+28 | ND |
|  | CB44 | ND |
|  | CB52 | ND |
|  | CB101 | ND |
|  | CB138 | ND |
|  | CB153 | ND |
|  | CB180 | 0.024 |
|  | CB183 | 0.036 |
|  | CB194 | 0.063 |
|  | CB209 | ND |
| PBDEs  (ng/g dw) | BDE85 | ND |
|  | BDE154 | 0.000 |
|  | BDE153 | 0.000 |
|  | BDE183 | ND |
|  | BDE202 | 0.001 |
|  | BDE201 | 0.001 |
|  | BDE204 | 0.001 |
|  | BDE197 | 0.001 |
|  | BDE(198+199+200+203) | 0.000 |
|  | BDE196 | 0.001 |
|  | BDE205 | 0.000 |
|  | BDE194 | 0.002 |
|  | BDE195 | 0.001 |
|  | BDE208 | 0.003 |
|  | BDE207 | 0.004 |
|  | BDE206 | 0.002 |
|  | BDE209 | ND |
|  | BDE47 | ND |
|  | BDE66 | ND |
|  | 2-MeO-BDE68 | ND |
|  | 2-MeO-BDE47 | ND |
|  | BDE100 | ND |
|  | BDE99 | 0.001 |
| ΣDDTs (ng/g dw) |  | 0.146 |
| ΣOCPs  (ng/g dw) |  | 0.466 |
| ΣPCBs  (ng/g dw) |  | 0.123 |
| ΣPBDEs (ng/g dw) |  | 0.018 |

*ND* not detected, dw dry weight
